# Supplementary material for: Harnessing synthetic active particles for physical reservoir computing
Source: Nat Commun. 2024 Jan 29;15:774. doi: 10.1038/s41467-024-44856-5 (PMC10825170; doi:10.1038/s41467-024-44856-5)
Supplement: Supplementary file 1 — Supplementary Information [file 41467_2024_44856_MOESM1_ESM.pdf]

Supplementary Information

Physical Reservoir Computing with Noisy Microswimmers

Xiangzun Wang[1,2], **Frank Cichos**[1*]

[1] *Peter Debye Institute for Soft Matter Physics, Molecular Nanophotonics Group, Universität Leipzig, 04103 Leipzig, Germany*
[2] *Center for Scalable Data Analytics and Artificial Intelligence (ScaDS.AI) Dresden/Leipzig, 04105 Leipzig, Germany*
[*] *To whom correspondence should be addressed; E-mail: cichos@physik.uni-leipzig.de*

Supplementary Notes

Contents

1 Effective Potential of θ **1**

2 Configuration of the RC **2**
2.1 RC architecture . . . 2
2.2 Time structure of the RC . . . 3
2.3 Default parameters of RC and optimization . . . 4
2.4 Binary and multi-value $\mathbb{W}_{\text{in}}$ . . . 4
2.5 Mackey–Glass series generation . . . 5

3 Normalized Root-Mean-Square Error (NRMSE) **5**

4 Prediction of Other Nonlinear Series **5**
4.1 Trigonometric function prediction . . . 5
4.2 Lorenz series prediction . . . 7

5 Estimation of the Signal-to-Noise Ratio **7**

6 RC Performances with Noises and Different Feedback Delays **8**

7 Experimental Setup **8**
7.1 Delay of the feedback loop . . . 9

8 Robustness of the RC System **9**
8.1 RC performance versus noise strength . . . 10
8.2 Robustness to perturbation . . . 10

1 Effective Potential of θ

Theoretically, the dynamics of θ of the active particle under a delayed attraction to an immobile target particle can be approximately interpreted as an over-damped quantity in an effective quartic potential $U_{\text{eff}}(\theta)$,

$$U_{\text{eff}}(\theta) = \frac{1}{\delta t}\left[\left(\frac{R_0}{v_0 \delta t} - 1\right)\theta^2 + \frac{1}{12}\theta^4\right]. \tag{1}$$

More details are elaborated in our previous work[1] and the corresponding Supplementary Information. The form of $U_{\text{eff}}(\theta)$ is determined by a dimensionless control parameter $v_0\delta t/R_0$, with v_0 the active particle speed and δt

the time delay. The constant $R_0 = a_{\text{active}} + a_{\text{imm}}$ is the minimal distance between the active and the target particle when the two particles physically contact. Supplementary Figure 1A depicts the theoretical potential U_{eff} as function of θ and $v_0\delta t/R_0$. As $v_0\delta t/R_0$ increases, the potential $U_{\text{eff}}(\theta)$ transitions from single well to double well shape at the transition point. The local minima of $U_{\text{eff}}(\theta)$ (red curves in Supplementary Figure 1A) represent the stationary rotational states of the active particle. With the increasing of $v_0\delta t/R_0$, the θ of the stationary state bifurcates from zero to two opposite values θ_+ and θ_-.

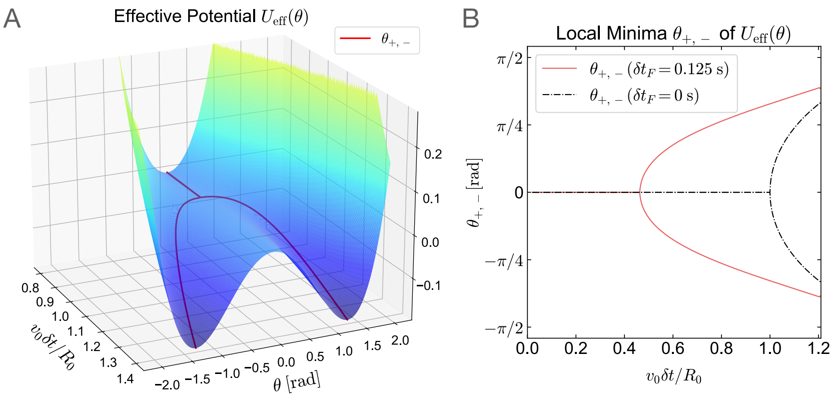

Supplementary Figure 1: Effective potential of θ of a delayed active particle node. **A**. Theoretical effective potential U_{eff} as function of θ and $v_0\delta t/R_0$ with the feedback delay $\delta t_F = 0$. The red curves denote the local minima of U_{eff}, i.e., the stationary rotational states of θ, marked as θ_+ and θ_-. $U_{\text{eff}}(\theta)$ transitions from single well to double well shape at $v_0\delta t/R_0 = 1$. **B**. $\theta_{+,-}$ as function of $v_0\delta t/R_0$ with δt_F of 0 s (black dashed curves) and 0.125 s (red solid curves). $\theta_{+,-}$ bifurcates from zero to two opposite values at the transition point of $U_{\text{eff}}(\theta)$. The results are plotted with a delay $\delta t = 0.6$ s and a varying v_0.

In real experiments, the feedback loop has a non-zero delay $\delta t_F > 0$, which causes a deviation between the detected and actual particle positions. The actual distance d between the laser and the active particle center also deviates from the programmed value $d_0 = 0.87$ µm. The propulsion speed v_0 of a particle is a function of d as studied in our previous work[2]. The angular speed of the active particle in the deterministic case is then

$$\omega(t) = \frac{v_0(d)}{R_0}\frac{f}{d} \tag{2}$$

with

$$\begin{aligned} f &= R_0 \sin(\theta_F) + d_0 \sin(\theta + \theta_F) \\ g &= d_0 \cos(\theta + \theta_F) + R_0 \cos(\theta_F) - R_0 \\ d &= (f^2 + g^2)^{\frac{1}{2}} \end{aligned} \tag{3}$$

and

$$\theta(t) = \int_{t-\delta t-\delta t_F}^{t-\delta t_F} \omega(t')\mathrm{d}t' \,, \quad \theta_F(t) = \int_{t-\delta t_F}^{t} \omega(t')\mathrm{d}t'. \tag{4}$$

In this case, $U_{\text{eff}}(\theta)$ is dependent not solely on $v_0\delta t/R_0$, but also complicatedly on d and δt_F. Therefore, the transition curves in Fig. 5**c**, **d** of the main text do not coincide with the $v_0\delta t/R_0$ contour lines.
Assuming ω as a constant, the solutions of θ in Eq. 2 denote the stationary rotational states $\theta_{+,-}$. Supplementary Figure 1B depicts the $\theta_{+,-}$ with the feedback delay δt_F of 0.025 and 0.125 s. Over the transition point, the distance $|\theta_+ - \theta_-|$ between the double wells of $U_{\text{eff}}(\theta)$ increases with $v_0\delta t/R_0$.

2 Configuration of the RC

2.1 RC architecture

Consider a reservoir with N_{node} physical nodes, the input layer for the n-th computation step is constructed as

$$\mathbb{U}_n = \mathbb{W}_{\text{in}}[b_{\text{in}}; \mathbf{X}_n] \tag{5}$$

with $[\cdot\,;\,\cdot]$ denoting a vertical concatenation. The vector $\mathbf{X}_n \in \mathbb{R}^{N_\mathrm{X}}$ is the input signal with a dimension of N_X. $\mathbb{W}_\mathrm{in} \in \mathbb{R}^{P_\mathrm{in}\times N_\mathrm{node}\times(1+N_\mathrm{X})}$ is the input weight matrix. The input bias b_in is a constant scalar. $\mathbb{U} \in \mathbb{R}^{P_\mathrm{in}\times N_\mathrm{node}}$ is the collection of the input layers $\mathbf{u}$ of the individual physical nodes

$$\mathbb{U}_n = [\mathbf{u}_n^1,\, \mathbf{u}_n^2,\, \cdots,\, \mathbf{u}_n^{N_\mathrm{node}}] \tag{6}$$

with the upper indices indicating the node number. Each input layer of a node $\mathbf{u}^i \in \mathbb{R}^{P_\mathrm{in}}$ is a vector containing P_in elements. These elements are sequentially input into the corresponding active particle as $u(T)$ as stated in the main text.

For each computation step, the virtual node states $\theta(T)$ of the physical nodes in the past P_out time steps are concatenated in an array of $\mathbf{\Theta}_n \in \mathbb{R}^{(P_\mathrm{out}\cdot N_\mathrm{node})}$,

$$\mathbf{\Theta}_n = [\theta^1(0), \theta^2(0)\cdots, \theta^{N_\mathrm{node}}(0), \theta^1(-1), \cdots \theta^{N_\mathrm{node}}(1-P_\mathrm{out})]. \tag{7}$$

T is an integer denoting the time step in a discrete time system with value zero representing the time of the current computation step. The virtual node states are contained in a vector $\mathbf{V}_n \in \mathbb{R}^{(1+N_\mathrm{X}+P_\mathrm{out}\cdot N_\mathrm{node})}$ together with the output bias b_out and the input $\mathbf{X}_n$,

$$\mathbf{V}_n = [b_\mathrm{out}; \mathbf{X}_n; \mathbf{\Theta}_n]. \tag{8}$$

The $\mathbf{V}$ of all computation steps in the training phase are collected in $\mathbb{V} \in \mathbb{R}^{(1+N_\mathrm{X}+P_\mathrm{out}\cdot N_\mathrm{node})\times L_\mathrm{train}}$,

$$\mathbb{V} = [\mathbf{V}_1, \mathbf{V}_2, \cdots, \mathbf{V}_{L_\mathrm{train}}], \tag{9}$$

where the lower indices denote the RC step, and L_train are the training steps.

Implementing the off-line training, the output weight matrix $\mathbb{W}_\mathrm{out} \in \mathbb{R}^{N_\mathrm{Y}\times(1+N_\mathrm{X}+N_\mathrm{node}\cdot P_\mathrm{out})}$ is derived by a ridge regression after the training phase,

$$\mathbb{W}_\mathrm{out} = \mathbb{Y}^\mathrm{target}\mathbb{V}^T(\mathbb{V}\mathbb{V}^T + \lambda\mathbf{I})^{-1}, \tag{10}$$

where λ is the regularization coefficient to avoid the over-fitting. $\mathbf{I}$ is the identity matrix. $\mathbb{Y}^\mathrm{target} \in \mathbb{R}^{N_Y\times L_\mathrm{train}}$ is the collection of the target signals during the training phase,

$$\mathbb{Y}^\mathrm{target} = [\mathbf{Y}_1^\mathrm{target}, \mathbf{Y}_2^\mathrm{target}, \cdots, \mathbf{Y}_{L_\mathrm{train}}^\mathrm{target}], \tag{11}$$

with each step signal $\mathbf{Y}_n^\mathrm{target} \in \mathbb{R}^{N_Y}$ as a vector with a dimension of N_Y. After the training, the output is derived with the trained $\mathbb{W}_\mathrm{out}$,

$$\mathbf{Y}_n = \mathbb{W}_\mathrm{out}[b_\mathrm{out}; \mathbf{X}_n; \mathbf{\Theta}_n]. \tag{12}$$

2.2 Time structure of the RC

For the series prediction task in the experiment, each computation cycle of the RC contains a 740 steps supervision period and a 400 steps free-running prediction period (each step lasts for P_in time steps due to the time multiplexing).

During the supervision period, the RC input is fed by an external signal $\mathbf{X}(n) = \mathbf{S}(n)$. The first 100 steps are the initiation period. The vectors $\mathbf{V}$ (Eq. 8) of these steps are not used for the $\mathbb{W}_\mathrm{out}$ calculation, in order to relieve the initial transient[3]. The next 600 steps are the training phase. After that, $\mathbb{W}_\mathrm{out}$ is calculated during the following waiting period of 40 steps (4 seconds in the experiment). $\mathbb{W}_\mathrm{out}$ is calculated using the ridge regression with the $\mathbf{V}$ of the steps in the training phase. The target is the series one step forward of the external signal $\mathbf{Y}^\mathrm{target}(n) = \mathbf{S}(n+1)$. The time consuming regression runs in another CPU thread to prevent influences on the experiment. Simultaneously, the RC keeps running with external signal feedings during this period, in order to wait for the $\mathbb{W}_\mathrm{out}$ result.

The supervision period is followed by the free-running prediction phase, during which the RC runs autonomously. Each step output $\mathbf{Y}(n)$ is derived using the trained $\mathbb{W}_\mathrm{out}$, then set as the input of the next step $\mathbf{X}(n+1) = \mathbf{Y}(n)$ (self-feed). Supplementary Figure 2 sketches the time line of a computation cycle of the RC.

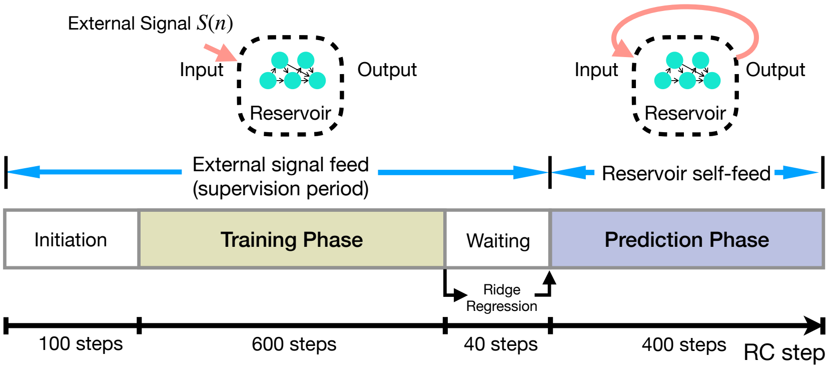

Supplementary Figure 2: The time line of a reservoir computation cycle in the experiments.

2.3 Default parameters of RC and optimization

The standard RC framework is not provided with training of its parameters or hyper-parameters except $\mathbb{W}_{\text{out}}$. However, the choices of them considerably affect the performance, especially for RCs with small reservoir sizes[4]. We optimize the configurations of the RC in simulations for specific tasks to acquire the best performances, then apply them in experiments.

If not mentioned, the parameters in this work are set with the following default values: number of physical nodes $N_{\text{node}} = 10$; number of input layer elements $P_{\text{in}} = 2$; number of virtual nodes for output $P_{\text{out}} = 400$; bias for output $b_{\text{out}} = 0.2$; regularization $\lambda = 10^{-5}$; speed of the active particle $v_0 = 2.78$ µms^{-1}; the minimal distance between the active and immobile particle $R_0 = a_{\text{act}}+a_{\text{imm}} = 1.1$ µm $+$ 1.5 µm $= 2.6$ µm; sampling period of the discrete time system $\Delta t = 0.05$ s; delay of active particle in the motion rule $\delta t = 0.05$ s, or expressed as $\delta T = 1$ $[\Delta t]$ in the discrete-time system. In simulations, the delay of the feedback loop $\delta t_F = 0.125$ s is considered. By default, the elements of $\mathbb{W}_{\text{in}}$ are chosen from $\{-2, 2\}$ by the pseudo-random module of Python (v.3.8.12, Numpy v.1.20.3) with a given seed. $\mathbb{W}_{\text{in}}$ and b_{in} are optimized by sweeping the seed from 0 to 200 and sweeping b_{in} through $\{0.02, 0.1, 0.2\}$ to find the lowest NRMSE of 200 RC steps results.

2.4 Binary and multi-value $\mathbb{W}_{\text{in}}$

The elements of the input weight matrix $\mathbb{W}_{\text{in}}$ (referred to as a mask in continuous physical RC applications[5]) is usually selected randomly from a uniform distribution over the same interval[3]. For instance in the range $[-2, 2]$,

$$\mathbb{W}^i_{\text{in}} \in \{-2, -2+4\frac{1}{N_{\text{rand}}-1}, ..., -2+4\frac{N_{\text{rand}}-2}{N_{\text{rand}}-1}, 2\}, \tag{13}$$

with $N_{\text{rand}} > 1$ the number of values for selection. In particular, $N_{\text{rand}} = 2$, i.e., a binary $\mathbb{W}_{\text{in}}$ is proven sufficient[4,5]. It is also reported that a $\mathbb{W}_{\text{in}}$ with more values helps to suppress the noise caused by quantization in the output layer[6].

We investigate the relation between N_{rand} and the performance of our RC for the Mackey–Glass series (MGS) prediction in simulations. For each value of N_{rand}, we firstly optimize $\mathbb{W}_{\text{in}}$ and b_{in} in a deterministic simulation (see Supplementary Note 2.3) to find out the lowest NRMSE, as depicted in Supplementary Figure 3A. $N_{\text{rand}} = \infty$ represents a uniform random distribution of $\mathbb{W}^i_{\text{in}}$ in $[-2, 2]$. The optimized configurations are used in simulations with Brownian motion ($D = 0.08$ µm^{2}s^{-1}). The resulting NRMSEs are shown in Supplementary Figure 3B.

In the deterministic simulation, the RC with $N_{\text{rand}} = 8$ exhibits the best performance, and that with the binary input weights $N_{\text{rand}} = 2$ shows the second best. For the RC with noise, the binary $\mathbb{W}_{\text{in}}$ yields the lowest NRMSE, which is 9.3% smaller than that with the uniform distribution ($N_{\text{rand}} = \infty$), thus behaves with the best resistance to noise. Therefore, binary input weights $\mathbb{W}^i_{\text{in}} \in \{-2, 2\}$ are chosen as default in this work. Note that the $\mathbb{W}^i_{\text{in}}$ with different N_{rand} in Supplementary Figure 3 are selected from the same range, thereby have different standard deviations of their distribution. This definition is different from that in[6], where the standard deviation of the $\mathbb{W}^i_{\text{in}}$ distribution is fixed.

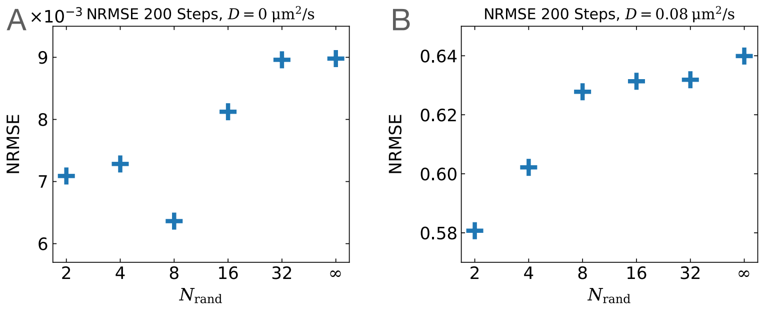

Supplementary Figure 3: NRMSE of the predictions as function of N_{rand} in the simulations. A. NRMSE of 200 steps Mackey–Glass series prediction as function of N_{rand} in deterministic simulation. $\mathbb{W}_{\mathrm{in}}$ and b_{in} are optimized for each N_{rand}. **B**. NRMSE of 200 steps predictions from simulations considering Brownian motion of active particles with a diffusivity of $D = 0.08\ \mu\mathrm{m}^2/\mathrm{s}$. Each data point is evaluated with 50 repeated predictions. RC configurations for each point are identical to the corresponding cases in panel A. $N_{\mathrm{rand}} = \infty$ denotes the uniform random distribution of $\mathbb{W}^i_{\mathrm{in}}$ in $[-2, 2]$.

2.5 Mackey–Glass series generation

The Mackey–Glass series used in this work is generated by the delay differential equation

$$\frac{\mathrm{d}S(n)}{\mathrm{d}n} = \alpha \frac{S(n-\tau)}{1 + S(n-\tau)^\beta} - \gamma S(n) \tag{14}$$

with $\alpha = 0.2$, $\beta = 10$, $\gamma = 0.1$, $\tau = 17$, initial condition $S(0) = 1.2$. The series is obtained by solving the equation numerically with the 4th order Runge–Kutta method and step size $\Delta n = 0.01$. The series is then re-sampled to $\Delta n = 1$ and shifted by -1 along the y-axis and then passed through a tanh function.

3 Normalized Root-Mean-Square Error (NRMSE)

To estimate the performance of the RC output, the normalized root-mean-square error (NRMSE) is calculated as

$$\mathrm{NRMSE} = \sqrt{\frac{\sum_{m=1}^{M} \sum_{n=1}^{N} \left(Y_m(n) - Y^{\mathrm{target}}(n)\right)^2}{M \sigma^2_{\mathrm{target}}}} \tag{15}$$

assuming a one-dimensional output Y. M denotes the total number of the independent repetition of the same computation. In deterministic simulation, $M = 1$. $Y_m(n)$ is the output trace of one of the repetitions, numbered by the lower index m. $Y^{\mathrm{target}}(n)$ is the target signal. n denotes the RC step, and N is the total number of steps for performance evaluation. σ_{target} is the standard deviation of the target Y^{target}. For multi-dimensional output $(x, y, \cdots)$, the total NRMSE is the root-mean-square of those of the individual dimensions

$$\mathrm{NRMSE} = (\mathrm{NRMSE}_x^2 + \mathrm{NRMSE}_y^2 + \cdots)^{\frac{1}{2}}. \tag{16}$$

4 Prediction of Other Nonlinear Series

4.1 Trigonometric function prediction

Besides the Mackey–Glass series (MGS), we also test our RC in the experiment with the free-running prediction of a non-chaotic periodic series, which is generated by a pure trigonometric function

$$S(n) = 0.2\big(\sin(0.1n)\cos(0.5n) + \sin(0.3n)\big). \tag{17}$$

Supplementary Figure 4 demonstrates the prediction results from the simulation and the experiment. The RC configuration is the same as that for the MGS prediction (Supplementary Note 2.3). The SNR is estimated in the simulation (Supplementary Note 5) as 1.83 (2.62 dB).

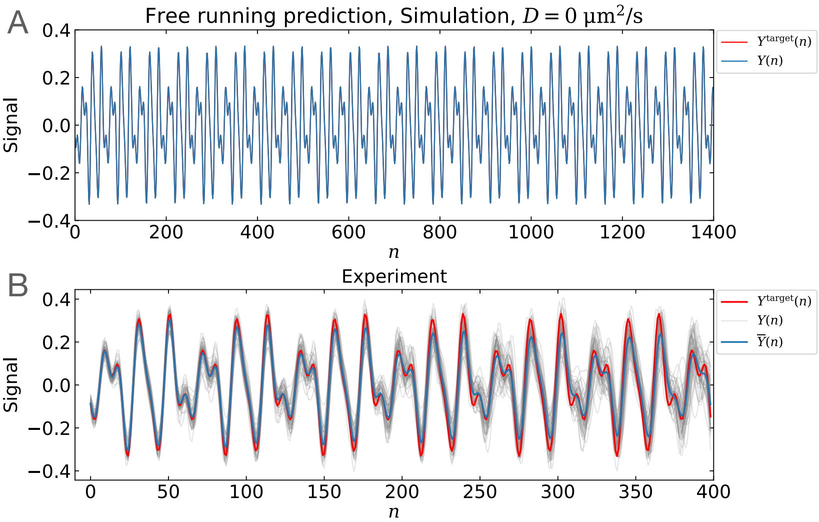

Supplementary Figure 4: Free running predictions of the trigonometric series. **A**. Result from a deterministic simulation. The prediction curve (blue) overlaps the target (red) due to a perfect matching. **B**. Results from the experiments. The gray curves denote the output traces from 50 repeated predictions. The blue curve denotes the mean of the output traces. $P_{\text{out}} = 200$. The other parameters are identical to those for Mackey–Glass series prediction (Supplementary Note 2.3).

As compared to the chaotic MGS, the prediction of the trigonometric series (TS) is apparently easier. In the deterministic simulation (Supplementary Figure 4A), the free running prediction is almost identical to the target with an extremely low NRMSE of 4.6×10^{-6} in 1400 steps. In the experiment (Supplementary Figure 4B), the RC outputs are disturbed by the particle Brownian motion (gray curves). Whereas the mean of the output traces $\overline{Y}$ can reproduce the main peaks and the period of the target signal. The minor peaks can be observed in $\overline{Y}$ up to 300 steps.
With the identical experimental environment, the NRMSE_{TS} of 200 steps TS prediction is 0.286, which is 66% lower than that of MGS $\text{NRMSE}_{\text{MGS}} = 0.853$ (Fig. 4**f** of the main text, $P_{\text{out}} = 200$). The results indicate that the TS prediction is also less impacted by noise than the MGS prediction. The presumable reason of the difference is that the effect of the noise is amplified due to the chaotic property of the Mackey–Glass system[7].

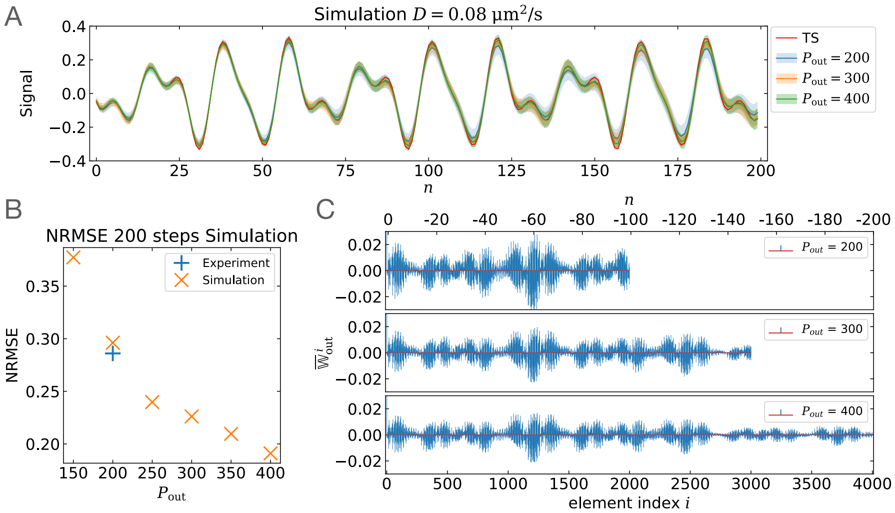

Supplementary Figure 5: Results of the trigonometric series prediction from stochastic simulation. **A**. Prediction results with P_{out} from 200 to 400, repeated 50 times for each case. The dash-dotted curves denote the mean of the output traces, and the corresponding colored areas denote the standard deviations of the outputs. **B**. NRMSE of 200 steps RC outputs versus P_{out} evaluated by 50 repeated predictions for each data point, compared with experimental results. **C**. Mean of the trained $\mathbb{W}_{\text{out}}$ for different P_{out}.

As shown in the simulation results with noise (Supplementary Figure 5A, B), the NRMSE decreases as the P_{out} increases. Hence for this task, utilizing historical reservoir states for the output is also an effective method for noise suppression, as the implemented in the main text for the MGS prediction task. The means of the trained output weights $\overline{\mathbb{W}}_{\text{out}}$ are plotted in Supplementary Figure 5C. The highest peak of $\overline{\mathbb{W}}_{\text{out}}$ appears at $n = -\,60$, corresponding to the fundamental period of TS.

4.2 Lorenz series prediction

We test our RC with the free running prediction of the 3-dimensional chaotic Lorenz series (LS), which is generated by

$$\begin{aligned} \dot{x} &= 10(y - x) \\ \dot{y} &= 28x - y - xz \\ \dot{z} &= xy - 2.667z \end{aligned} \tag{18}$$

with step $\mathrm{d}t = 0.01$, then re-sampled to $\mathrm{d}t = 0.02$ and scaled by $1/30$. Supplementary Figure 6 shows the simulation results of the LS prediction with and without noise.

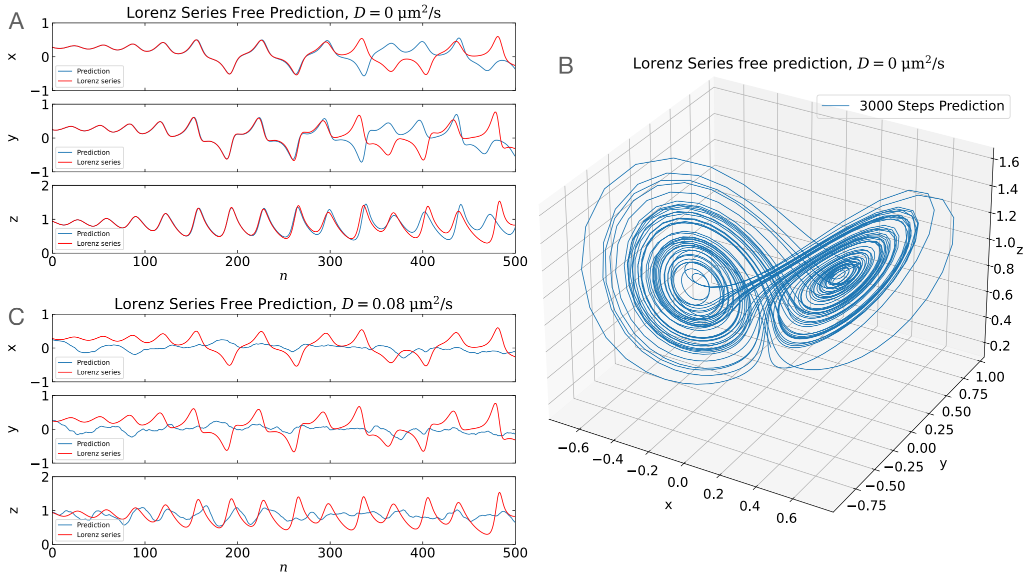

Supplementary Figure 6: Simulation results of the free running predictions of the 3-dimensional Lorenz series. Simulations were conducted without (**A**, **B**) and with noise (**C**) on the nodes. **A**, **C**. Target (red lines) and prediction (blue lines) in three components. **B**. Phase diagram of the prediction. RC configuration: $N_{\text{node}}= 10$, $P_{\text{in}} = 10$, $P_{\text{out}} = 400$, $b_{\text{in}} = b_{\text{out}} = 0.5$, $\mathbb{W}^{i}_{\text{in}} \in [-1, -1/3, 1/3, 1]$, regularization $\lambda = 10^{-9}$. The other parameters are set as default (Supplementary Note 2.3).

The simulation results without noise display a decent prediction of the LS in 250 steps (5.52 Lyapunov time, Supplementary Figure 6A) with a NRMSE of $6.2{\times}10^{-2}$, and the iconic butterfly pattern in the phase diagram up to 3000 steps (Supplementary Figure 6B). However, the results with noise do not show effective predictions, as plotted in Supplementary Figure 6C. The reason of that is presumed to be the high degree of chaos of the Lorenz system. The maximal Lyapunov exponents of the LS is $\lambda_{\text{LS}} \approx 0.906$, which is much higher than that of MGS, $\lambda_{\text{MGS}} \approx 0.006$[8]. The noise in the former thereby induces much larger impact in the RC system than the latter.

5 Estimation of the Signal-to-Noise Ratio

The noise caused by the particle Brownian motion affects directly the angular position ϕ of the active particle, but not directly the input signal u or the node state θ, as shown in Eq. 3–5 of the main text. To evaluate the signal-to-noise ratio (SNR) of our RC, simulations are conducted with the identical configuration as in the

experiment. The changes of the angular position $\Delta\phi(T) = \phi(T) - \phi(T-1)$ of each time step are measured with noise ($\Delta\phi_N$) and without noise ($\Delta\phi_0$). The SNR is evaluated as

$$\text{SNR} = \frac{\sum_T \Delta\phi_0^2(T)}{\sum_T \Delta\phi_N^2(T) - \sum_T \Delta\phi_0^2(T)}. \tag{19}$$

The quantity $\Delta\phi_N^2(T)$ is averaged by that from 100 repetitions of the same process. The SNR is evaluated by the data during the supervision period of the RC (see Supplementary Note 2.2), resulting a SNR = 1.9 (2.79 dB).

6 RC Performances with Noises and Different Feedback Delays

Supplementary Figure 7 plots the performance of the RC as function of v_0/R_0 and δt for the prediction of Mackey–Glass series. The results are evaluated by simulations with the Brownian motion on active particles considered. Supplementary Figure 7A, B correspond to the deterministic simulation results in Fig. 5**c**, **d** of the main text. The correlations between the normalised root-mean-square error (NRMSE) of the predictions and $v_0\delta t/R_0$ are similar as that in deterministic simulations, whereas larger NRMSE can be observed with higher velocity v_0. This effect is presumably caused by the feedback delay δt_F (Supplementary Note 7.1). δt_F causes the deviation between the actual and detected particle position, according to the latter the laser is deployed. This position deviation results in a deviation between the expected and real particle propulsion directions (Supplementary Figure 8B), and contains the contribution of the particle Brownian motion during δt_F. The impact of the Brownian motion is amplified by the particle speed v_0. As a proof of the above interpretation, Supplementary Figure 7C depicts the RC performance with $\delta t_F = 0.025$ s. In contrast to Supplementary Figure 7A with $\delta t_F = 0.125$ s, the NRMSE in Supplementary Figure 7C does not show the increasing trend with v_0.

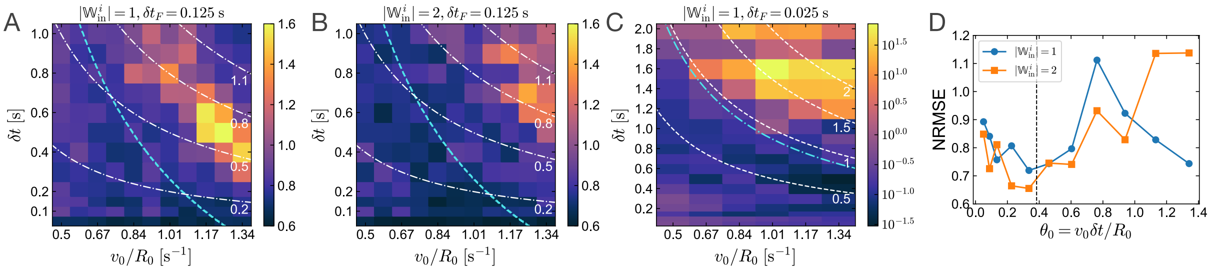

Supplementary Figure 7: RC performance as function of v_0/R_0 and δt of the physical node with noise. Each grid point denotes the NRMSE evaluated by 50 repeated Mackey–Glass series 200-step-predictions in the simulations. Brownian motion of the active particles are taken into account as noise of the RC. The white dash-dotted curves denote the contour lines of $v_0\delta t/R_0$. The cyan dashed curves denote the theoretical transition points of $U_{\text{eff}}(\theta)$ from single well to double well shape. $\mathbb{W}_{\text{in}}$ and b_{in} are optimized for each grid point. The elements of $\mathbb{W}_{\text{in}}$ are selected from $\{-1, 1\}$ (**A, C**) and $\{-2, 2\}$ (**B**). The feedback delay $\delta t_F = 0.125$ s (**A, B**) and 0.025 s (**C**). The parameters of the RCs in (**A**) and (**B**) are identical to that in Fig. 5**c** and **d** of the main text, respectively. **D**. Comparison of the NRMSE results of the diagonal grid points in panel A and B (from lower left to upper right) as function of θ_0. The dashed line denotes the transition point of U_{eff}.

7 Experimental Setup

Fig. 1**a** of the main text illustrates the experimental setup used for this work, including a feedback loop for active swimmer manipulation. A laser of 532 nm wavelength from the module (CNI, MGL-H-532-1W) is expanded in the beam diameter by two lenses with 35 mm and 150 mm focal lengths. The laser beam is then projected to a high-speed reflective Spatial Light Modulator (SLM, Meadowlark Optics, HSP512-53), which modulates the phase of the laser by a nematic liquid crystal on silicon (LCoS) pixels in arrays. The phase pattern of the SLM is controlled by an input signal from a PC. The reflected beam with the modulated wavefront is guided by mirrors through two tube lenses (500 mm, 300 mm focal lengths) and a mask in between to an inverted microscope (Olympus, IX73). The mask is a small opaque dot painted on the glass window located at the focal point of the 500 mm lens, blocking the unmodulated laser reflected by the surface of the SLM. Inside the microscope, the laser is reflected by a dichroic beam splitter (Omega Optical, 560DRLP), then focused by an objective (100x,

Olympus, UPlanFL N x100/1.30, Oil Iris, NA 0.6–1.3) on the sample plane. The full width at half maximum (FWHM) of the focus is about 0.6 µm. The laser on the sample is manipulated by the phase pattern of the SLM. Before each experiment, the feedback loop is calibrated by scanning a laser point on a sample of a thin layer of Nile Blue fluorescent dye, in order to establish the relation between the laser focus position and the phase pattern of the SLM.
The sample is illuminated by white light from an LED lamp (Thorlabs, SOLIS-3C) through an oil-immersion dark field condenser (Olympus, U-DCW, NA 1.2–1.4). The light enters the sample with a glancing angle, so only the rims of the particles in the sample are illuminated. The particles are observed as bright rings in the view of the microscope (Fig. 1**d** of the main text and Supplementary Movie 1). The image of the sample is projected through the objective lens and one tube lens (180 mm focal length) inside the microscope, then two tube lenses (100 mm, 150 mm focal lengths) to a camera (Hamamatsu digital sCMOS, C11440-22CU). The numerical aperture (NA) of the objective is set to a value below the minimal NA of the dark-field condenser. Two filters (EKSMA Optics 246-2506-532, Thorlabs FESH0800) in front of the camera block the back reflections of the laser from the sample glass and other optics. The camera applies a pixel binning of four to reduce the image size and processing speed, resulting in a sample resolution of 0.086 µm/pix.
The LabVIEW program (v. 2019) on a desktop PC (Intel(R) Core™ i7-7700K CPU @4×4.20 GHz) controls the feedback loop. The program receives and analyzes the image from the camera and also measures and records the particle positions. According to the rule of motion described in the main text, LabVIEW calculates the desired moving directions of the particles, then the corresponding laser positions for particle propulsion. LabVIEW calculates the corresponding phase pattern on the SLM with the help of a graphics card (NVIDIA GeForce GTX 1050Ti) and the CUDA Toolkit and then sends the pattern to the SLM. The camera takes images with a sampling period of $\Delta t = 50$ ms. The feedback loop is updated with the same rate.
The LabVIEW program is also responsible for the operation of the reservoir computer (RC). During the training phases, LabVIEW records and collects the delay angles $\theta(T)$ of the active particles as virtual node states (see the main text), and calculates the output weights $\mathbb{W}_{\text{out}}$ via a ridge regression (Eq. 10) when the training phase finishes. In order to keep the experiment fluently running simultaneously, the ridge regression is executed by another CPU thread with a Python node (Python v. 3.6.5, Numpy v. 1.19.5). In the prediction phase, the output is derived for each RC step by a matrix multiplication according to Eq. 12. The time structure of the RC cycle is described in Supplementary Note 2.2.

7.1 Delay of the feedback loop

The time for signal transfer, data processing, and the sampling period causes an inevitable systematical latency in the feedback loop. The time line of the signal processing is sketched in Supplementary Figure 8A. A feedback delay δt_F of about 100∼130 ms from the exposure of the camera to the update of the laser exists in the experiment. The time consumption by LabVIEW increases from around 10 ms to 40 ms during one RC cycle (Supplementary Figure 2), because the increasing number of the recorded node states in the PC memory slows down the processing speed. δt_F was determined in the experiment with a sample of Nile Blue. LabVIEW changes the laser position alternatively, then measures in which frame the change can be observed by the camera. By adjusting the camera frame interval, δt_F can be accurately determined with frames of lag and the sampling period.
Because of the feedback delay, the particle position detected by the camera ($\mathbf{r}(t - \delta t_F)$) deviates from its actual position ($\mathbf{r}(t)$). In the experiment, this deviation contains the contribution of the particle Brownian motion during δt_F. Since the laser is deployed according to the detected particle position, the actual particle propulsion induced by the laser also differs from the expected direction. Supplementary Figure 8B sketches the effect of the feedback delay on the dynamics of the active particle. It is worth noting that the feedback delay makes the active particle dynamics much more complicated than that described by Eq. 3–5 in the main text (see Supplementary Note 1). However, as long as the nonlinearity and the fading memory of the physical node are satisfied, the RC remains functional.

8 Robustness of the RC System

We investigate the robustness of our RC system for the free running prediction as function of $n_{\text{hist}} = P_{\text{out}}/P_{\text{in}}$ with $P_{\text{in}} = 2$ in the simulations.

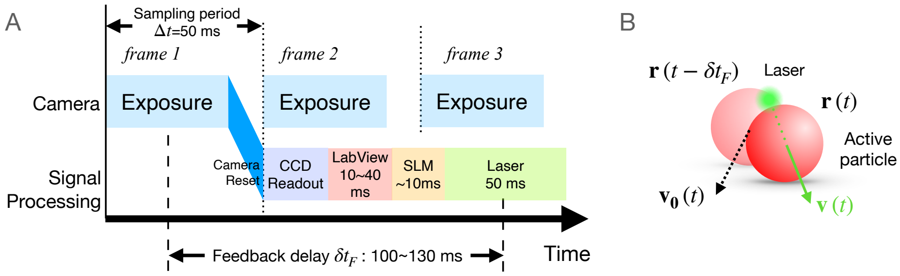

Supplementary Figure 8: Delay of the feedback loop. **A**. Time line of data processing of the feedback loop. The feedback delay δt_F is the latency time from the middle of the camera exposure to the middle of the laser illumination. **B**. Sketch of the impact of δt_F on the active particle dynamics. $\mathbf{r}(t)$ denotes the actual particle position. $\mathbf{r}(t-\delta t_F)$ is the detected particle position by the camera, according to which the laser (green dot) is allocated. The dashed arrow and $\mathbf{v}_0(t)$ represent the desired particle propulsion direction. The green solid arrow and $\mathbf{v}(t)$ denote its actual direction.

8.1 RC performance versus noise strength

Supplementary Figure 9A demonstrates the NRMSE of the RC free running predictions versus the strength of the noise in the RC. The noise strength is represented by the diffusivity D of Brownian motion of the active particles relative to that in the experiments $D_0 = 0.08\ \mu\text{m}^2\text{s}^{-1}$.

8.2 Robustness to perturbation

To test the robustness of the RC system for the free running prediction, a perturbation of $\epsilon = 10^{-4}$ is added into one step of the input $S(n)$ (Mackey-Glass series),

$$S^*(n=0) = S(n=0) + \epsilon. \tag{20}$$

The corresponding output $O^*(n)$ is measured and compared with the output without perturbation $O(n)$. Noises are not considered in this test. $n = 0$ denotes the starting step of the prediction phase (see Supplementary Note 2.2), so the training processes of the RCs for both cases are identical. Supplementary Figure 9B plots the deviation of the outputs $|O^*(n) - O(n)|$ relative to the perturbation ϵ with different n_{hist} of the RCs. To obtain a fair comparison, the RCs with different n_{hist} have the same $\mathbb{W}_{\text{in}}$ and b_{in}, which are not optimized for their prediction performances (see Supplementary Note 2.3).
To characterize the speed of the increment of the outputs deviation, the least RC steps Λ where the relative deviation exceeds the Euler's number e,

$$\frac{|O^*(\Lambda) - O(\Lambda)|}{\epsilon} > e \tag{21}$$

is defined, as plotted in Supplementary Figure 9C. The RCs with larger n_{hist} possesses higher Λ, i.e., obtain larger robustness to the perturbation.

Supplementary References

[1] X. Wang, P.-C. Chen, K. Kroy, V. Holubec, and F. Cichos, Spontaneous Vortex Formation by Microswimmers with Retarded Attractions, *Nat Commun* **14**, 56 (2023).

[2] M. Fränzl, S. Muiños-Landin, V. Holubec, and F. Cichos, Fully Steerable Symmetric Thermoplasmonic Microswimmers, *ACS Nano* **15**, 3434–3440 (2021).

[3] M. Lukoševičius, "A Practical Guide to Applying Echo State Networks", in *Neural Networks: Tricks of the Trade*, Vol. 7700, edited by G. Montavon, G. B. Orr, and K.-R. Müller (Springer Berlin Heidelberg, Berlin, Heidelberg, 2012), pp. 659–686.

[4] L. Appeltant, G. Van der Sande, J. Danckaert, and I. Fischer, Constructing Optimized Binary Masks for Reservoir Computing with Delay Systems, *Sci Rep* **4**, 3629 (2014).

[5] L. Appeltant, M. Soriano, G. Van der Sande, J. Danckaert, S. Massar *et al.*, Information Processing Using a Single Dynamical Node as Complex System, *Nat Commun* **2**, 468 (2011).

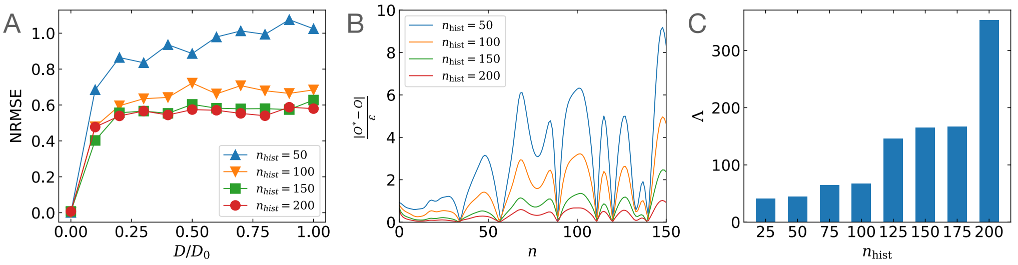

Supplementary Figure 9: Robustness tests of the RC system in the simulations. A. The NRMSE of 50 predictions as function of the noise strength in the RC with different $n_{\rm hist}$. The noise strength is represented by the diffusivity D of the Brownian motion of the active particles. D_0 denotes the diffusivity in the experiment. **B**. The relative deviation of the RC outputs with and without a perturbation ϵ in the inputs (O and O^* respectively). n denotes the RC step. The perturbation of $\epsilon = 10^{-4}$ is added at the starting step of the prediction phase $n = 0$. **C**. The least RC steps Λ when the relative output deviation exceeds the Euler's number e, i.e., $|O^*(\Lambda) - O(\Lambda)|/\epsilon > e$.

[6] M. C. Soriano, S. Ortín, D. Brunner, L. Larger, C. R. Mirasso *et al.*, Optoelectronic Reservoir Computing: Tackling Noise-Induced Performance Degradation, *Opt. Express* **21**, 12 (2013).

[7] P. Antonik, M. Haelterman, and S. Massar, Brain-Inspired Photonic Signal Processor for Generating Periodic Patterns and Emulating Chaotic Systems, *Phys. Rev. Applied* **7**, 054014 (2017).

[8] H. Jaeger and H. Haas, Harnessing Nonlinearity: Predicting Chaotic Systems and Saving Energy in Wireless Communication, *Science* **304**, 78–80 (2004).
