# Supplementary material for: Harnessing synthetic active particles for physical reservoir computing
Source: Nat Commun. 2024 Jan 29;15:774. doi: 10.1038/s41467-024-44856-5 (PMC10825170; doi:10.1038/s41467-024-44856-5)
Supplement: Supplementary file 3 — Description of Additional Supplementary Files [file 41467_2024_44856_MOESM3_ESM.pdf]

Description of Additional Supplementary Files

Supplementary Movie 1 Real time video of the sample during the reservoir computation in the experiment under the view of the dark-field microscope.
